# Use of ward closure to control outbreaks among hospitalized patients in acute care settings: a systematic review

**DOI:** 10.1186/s13643-015-0131-2

**Published:** 2015-11-07

**Authors:** Holly Wong, Katherine Eso, Ada Ip, Jessica Jones, Yoojin Kwon, Susan Powelson, Jill de Grood, Rose Geransar, Maria Santana, A. Mark Joffe, Geoffrey Taylor, Bayan Missaghi, Craig Pearce, William A. Ghali, John Conly

**Affiliations:** W21C Research and Innovation Centre, Cumming School of Medicine, University of Calgary, GD01 TRW Building, 3280 Hospital Drive NW, Calgary, Alberta Canada T2N 4Z6; Health Sciences Library, Libraries and Cultural Resources, University of Calgary, HSC 1450, Health Sciences Centre, 3330 Hospital Drive NW, Calgary, Alberta Canada T2N 4N1; Infection Prevention and Control, Alberta Health Services, #303 CSC, 10240 Kingsway, Edmonton, Alberta Canada T5H 3V9; Department of Medicine, Faculty of Medicine and Dentistry, University of Alberta, 2D3.05 WMC, Edmonton, Alberta Canada T6G 2B7; Department of Medicine, Cumming School of Medicine, 3280 Hospital Drive NW, Calgary, Alberta Canada T2N 4Z6; Snyder Institute for Chronic Diseases, 3280 Hospital Drive NW, Calgary, Alberta Canada T2N 4Z6; Department of Community Health Sciences, University of Calgary, 3280 Hospital Drive NW, Calgary, Alberta Canada T2N 4Z6; O’Brien Institute for Public Health, 3280 Hospital Drive NW, University of Calgary, 3280 Hospital Drive NW, Calgary, Alberta Canada T2N 4Z6

## Abstract

**Background:**

Though often used to control outbreaks, the efficacy of ward closure is unclear. This systematic review sought to identify studies defining and describing ward closure in outbreak control and to determine impact of ward closure as an intervention on outbreak containment.

**Methods:**

We searched these databases with no language restrictions: MEDLINE, 1946 to 7 July 2014; EMBASE, 1974 to 7 July 2014; CINAHL, 1937 to 8 July 2014; and Cochrane Database of Systematic Reviews, 2005 to May 2014. We also searched the following: IndMED; LILACS; reference lists from retrieved articles; conference proceedings; and websites of the CDCP, the ICID, and the WHO. We included studies of patients hospitalized in acute care facilities; used ward closure as a control measure; used other control measures; and discussed control of the outbreak(s) under investigation. A component approach was used to assess study quality.

**Results:**

We included 97 English and non-English observational studies. None included a controlled comparison between ward closure and other interventions. We found that ward closure was often used as part of a bundle of interventions but could not determine its direct impact separate from all the other interventions whether used in parallel or in sequence with other interventions. We also found no universal definition of ward closure which was widely accepted.

**Conclusions:**

With no published controlled studies identified, ward closure for control of outbreaks remains an intervention that is not evidence based and healthcare personnel will need to continue to balance the competing risks associated with its use, taking into consideration the nature of the outbreak, the type of pathogen and its virulence, mode of transmission, and the setting in which it occurs. Our review has identified a major research gap in this area.

**Electronic supplementary material:**

The online version of this article (doi:10.1186/s13643-015-0131-2) contains supplementary material, which is available to authorized users.

## Background

While significant progress has been made in preventing device and procedure-related healthcare-associated infections (HAI), the threat of antimicrobial resistant organisms (ARO) and *Clostridium difficile* continues. In the USA, the prevalence rate of HAI was 4 % in 2011 [1], and it has been estimated that there are at least two million ARO-related infections and 23,000 deaths each year [2], resulting in $26–$33 billion additional medical costs [3]. An estimated 220,000 HAI and 8000 related deaths occur in Canada per year [4]. Healthcare-associated *C. difficile* and vancomycin-resistant *Enterococci* infections increased from 2007 to 2012, and carbapenemase-producing organisms appeared in 2010 [5]. The cost of readmissions alone due to nosocomial *C. difficile*-associated diarrhea is estimated to be at least $128,200 CDN per year per facility [6]. These observations highlight the need for more effective prevention and control practices and better therapy.

Outbreaks of HAI in healthcare facilities are not only serious clinical events when affecting vulnerable patient populations but are highly disruptive to care delivery. Closure of affected clinical areas typically involves suspending new patient admissions and has been used as a means of controlling HAI outbreaks [7]. However, ward closures restrict patient access to necessary care, may lead to detrimental outcomes, and can be extremely expensive to implement. Consequently, the role of ward closure in outbreak control should be better understood.

Complete ward closures are typically exercised when other outbreak measures have failed, or in the setting of highly virulent organisms, or those known to spread rapidly [8]. However, whether ward closure is a necessary control intervention is not clearly established in the literature.

A number of studies have described the use of ward closure for the purpose of outbreak control. One systematic review of worldwide HAI epidemics published in the Worldwide Database of Nosocomial Outbreaks between 1965 and 2005 found that some level of ward closure was used in 194 outbreaks, with a median closure time of 14 days and closure rate of 12.4 % [8]. Geriatric units were significantly more likely to be closed due to outbreaks compared to pediatric wards, and infectious pathogens were significantly more likely to lead to ward closure compared to contaminated medical equipment. Two specific groups of pathogens were most often associated with ward closure: norovirus (for 44.1 % of ward closures) and influenza/parainfluenza virus (for 38.5 % of ward closures).

The literature generally suggests that ward closure is a necessary control intervention as part of a bundle [9–11] versus a bundle that does not include ward closure [12, 13]. However, an analysis of a large standardized data set from 2009–2012 from the Hospital Norovirus Outbreak Reporting Scheme in the UK found that in instances where no ward closure was used, the length of outbreaks was similar to those where wards were closed but with fewer patients and healthcare workers (HCW) affected (in total and per day of outbreak) [14].

To gain a better understanding of the role of ward closure in controlling outbreaks, we systematically reviewed the published academic literature examining the use and impact of ward closure for controlling outbreaks in the acute care hospital setting. In addition to this review, we developed a web-based environmental scan survey that was distributed to IP&C practitioners and physicians at acute care sites across Canada. The present systematic review had two objectives: (1) to identify studies that describe ward closure as an outbreak control measure in sufficient detail to determine how ward closure was defined and what was done and (2) to determine the impact of ward closure on outbreak control by answering the following question: In hospitalized patients of all ages, does the use of partial or complete hospital ward closure have a significant impact on the control of an outbreak due to invasive infection or colonization by pathogenic microbes with the potential for spread, as compared to not using hospital ward closure, with or without the use of other infection control interventions and/or practices? These two questions guided the protocol development, which then informed the screening and selection process.

## Methods

This review is not registered with PROSPERO.

### Search strategy and selection criteria

To identify relevant references for this review, we searched the following databases with no language restrictions or other limits: Ovid MEDLINE, including In-Process & Other Non-Indexed Citations, 1946 to 7 July 2014; Ovid EMBASE, 1974 to 7 July 2014; CINAHL Plus with Full Text, 1937 to 8 July 2014; and Cochrane Database of Systematic Reviews, 2005 to May 2014. Our search consisted of selected subject headings and keywords related to the use of ward closure, combined with terms for outbreaks of infectious diseases (see Additional file 1). We also searched IndMED, using the same keywords, and LILACS, using a combination of the keywords in English and some of their Spanish and Portuguese equivalents. In addition, we searched reference lists from retrieved articles and journals, conference proceedings, and the websites of the Centers for Disease Control and Prevention, the International Centre for Infectious Diseases, and the World Health Organization.

Two authors independently reviewed the title and abstract of all articles resulting from the searches and the retrieved full texts of the relevant articles. The reviewers appraised the published full-text articles for inclusion according to the five criteria described below; articles were rejected if they did not meet all of the criteria. Disagreements during title and abstract screening and full-text review were resolved through third-party adjudication.

Only those articles that were outbreak investigation studies of hospitalized patients at acute care hospitals/facilities, including teaching and specialized institutions, were included. Studies set in a long-term acute care hospital were also included; however, studies set in a long-term care facility, rehabilitative setting, or outpatient clinic at a tertiary acute care hospital/facility were excluded. To be included, studies needed to identify ward closure (complete or partial) for at least 48 h (or length not specified) as an intervention to help control outbreaks. We defined “complete ward closure” as the application of ward closure across all beds on a ward/unit and “partial ward closure” as the application of ward closure to some, but not all, of the beds on a ward/unit. “Ward closure” included any or all of the following: no new patients admitted to the area; no transfers to other units within the healthcare facility allowed unless required for ongoing care; and no transfers to other healthcare facilities, including long-term care, with no restrictions on discharge home [14]. “Ward closure” was also assumed if the following synonyms and word variants were used: “unit closure,” “wing closure,” “partial hospital closure,” “halt new admissions,” “partial hospital closure,” “no new admission,” “closure,” “limited admissions,” “delayed admissions,” and “department closure.” Studies were also included only if a comparison intervention or another infection control intervention other than ward closure was applied and if they discussed control of the outbreak(s) under investigation as an outcome. We adopted the Alberta Health Services definition of outbreak: “the perceived, or true occurrence of more cases of a communicable disease than expected in a given area, or among a specific group of people over a defined period of time” [15]. Measures of this outcome included narrative accounts of outbreak control, number of cases of illnesses, number of colonized or infected inpatients, attack rates, relapse rates, and number of deaths attributable to the causative pathogen. Only original research studies were included, but conference abstracts were reviewed for relevance; if an abstract was deemed relevant, the corresponding author was contacted by one of the librarians for the published full text. We also excluded studies that used surveys, secondary data analysis, non-original reports, grey literature, editorials, letters, cost analyses, and reviews.

### Data extraction and analysis

The included studies were systematically reviewed and relevant data was extracted from each article on the following parameters: study design, setting and population characteristics, causative pathogen(s), details of ward closure, details of other outbreak control interventions, outcomes relevant to the review, including the number of patients colonized and/or infected, and the role of ward closure for controlling the outbreak were extracted and recorded by one of the authors. Data from non-English full-text articles were extracted by a researcher who was a native or fluent speaker of the language and had knowledge of data extraction for systematic reviews. Relevant extracted data were collated in a descriptive summary and tabular format based on the findings from the parameters listed above.

We adopted Juni and colleagues’ [16] component approach to assess the quality of each study included in this review. Six evaluative criteria were adapted from components of the GRADE approach [17] and the Downs and Black checklist [18] to develop an aggregate measure for “confidence in the estimate of effect of the body of evidence,” as done by Hsu and colleagues [19] using GRADE. The first five criteria were taken from the Downs and Black’s checklist for measuring study quality and the sixth criterion was developed by the authors to assess the accuracy (reliability and validity) of the outcome measures: (1) Are the characteristics of the patients included in the study clearly described? (2) Is the intervention of interest clearly described? (3) Are the main findings of the study clearly described? (4) Were the main outcome measures used accurate (valid and reliable)? (5) Did the authors address the issue of confounding in the analyses from which the main findings were drawn? (6) Did the authors confirm cases using acceptable diagnostic methods? For each criteria, a score of “0” was assigned if the criteria was not met and “1” if the criteria was met, providing a summated score between 0 and 6 for each article, where 0–1 indicates very low quality; 2–3 indicates low quality; 4–5 moderate quality; and 6 indicates high quality.

## Results

**Fig. 1:**
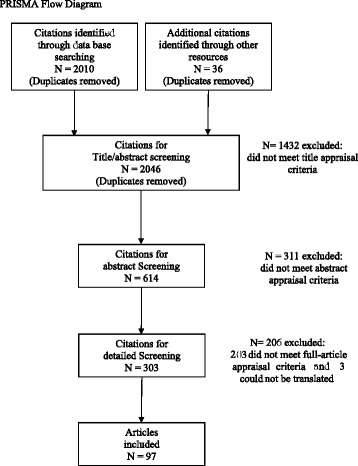
PRISMA flow diagram

From the 2095 references gathered from all the sources searched, a total of 97 English and non-English articles in Dutch, French, German, Japanese, and Spanish were accepted for inclusion (Fig. 1).

Of the 97 included studies, 67 were case series, 14 were case–control studies, 5 were cohort studies, 5 were before-and-after studies, 5 were interrupted time series studies, and 1 was a time series study. As there were no studies that included a controlled comparison between ward closure and other interventions, the studies included in this review only allowed us to fulfill our first objective. Thus, this review purely focused on studies that described how ward closure was used as an outbreak control intervention and its impact on the outbreak.

From the details provided within the context of the setting and population, the studies were organized firstly by the organ system(s) affected and secondly by the genus of the causative pathogen within each of these organ system categories. The organ system and genus categorization lent itself very well to an additional categorization by the mode of transmission, which is the basis for infection prevention and control precautions. The organ system categories included: “gastrointestinal,” consisting of 17 studies; “respiratory,” consisting of 11 studies; and “multiple/mixed,” which includes the central nervous system, skin/soft tissue, urinary tract, eye, abdominal, and vascular or when more than one system is affected simultaneously and consisting of 63 studies. Of the studies in the third category, eight studies described predominant colonization, 12 studies described predominant infection, and 43 studies described a combination of colonization and infection. A sixth category included six studies that described the impact of infection control policies and of specific interventions on outbreak control. The modes of transmission relevant to our study included contact (both direct person-to-person and indirect via fomites and inanimate objects), droplet (via large droplets within a 1–2 m radius of the individual), and airborne (via small droplet nuclei capable of spreading over distance of greater than 2 m through the air [20]).

### Gastrointestinal system (Table 1)

**Table 1:** Summary table for accepted studies—gastrointestinal system

	Setting (beds); country	Definition of ward closure (length)	Other measures	Inpatient outcomes (includes index case(s))^a^	Controlled (Y, N, NA)	Due to^b^
*Clostridium difficile*						
Cherifi et al. [28]	4 geriatric wards (97 total) at a teaching hospital (758); Belgium	No new admissions; no transfers (10d)	8	21/92 in total: 11 (52 %) died; 6 (29 %) relapsed	Y	All
Hastie et al. [23]	Urological ward; England	No new admissions (1m)	5	17/42 in total: all infected; 4 (24 %) relapsed	NA	Multiple
Ratnayake et al. [25]	Vascular acute surgery ward (24); Scotland	No new admissions (2w)	7	9 in total: 2 (22 %) died	Y	All
Norovirus						
Fretz et al. [22]	Internal medicine, intensive care, surgery, and orthopedics departments at a general hospital (176); Austria	No new admissions (3 occasions: 11d, 9d, 9d)	3	56 in total	NA	All
Hoffmann et al. [36]	34 wards at a teaching hospital; Germany	Limited transfers (6d)	3	116 in total	Y	NA
Kanerva et al. [34]	23 wards at a tertiary hospital (504); Finland	No new admissions; limited transfers	5	240 in total: 181 (75 %) positive; 9 (4 %) died	NA	Multiple
McCall and Smithson [31]	Acute elderly care ward; Ireland	No new admissions; no transfers; limited discharges (3d)	9	20 in total: 6 (30 %) positive, 14 (70 %) assumed	Y	All
Russo et al. [35]	3 extended care (30 each), acute care (37) wards at an elderly extended care facility (380); Australia	No new admissions; limited discharges; (2 occasions: 22d, 13d)	10	58 in total	N: seemed to limit the outbreak	Uncertain
Stevenson et al. [32]	11 wards at a geriatric hospital (300); England	Stage 1: unspecified closure	Stage 1: 4 Stage 2: 3 Stage 3: 1	95 in total	N: outbreak declared over but new cases	All
		Stage 2: no new admissions; no transfers; limited discharges (12d)				
Weber et al. [26]	Pediatric psychiatric unit (10) at a teaching hospital; USA	No new admissions (9d)	6	3/4 in total	Y	All
Zingg et al. [30]	2 internal medicine wards at a tertiary hospital (960); Switzerland	No new admissions; no transfers	6	16/115 in total: 12 (75 %) positive, 3 (19 %) assumed, 1 (6 %) symptomatic	N: reduced number of new cases	All
Rotavirus						
Clark et al. [21]	Infectious disease (10) and general infant (16) wards; England	No new admissions (5d)	3	20 in total	Y	All
Srinivasan et al. [37]	Neonatal unit; USA	Unspecified closure of transitional nursery	5	23/28 in total: 5 (22 %) positive; 18 infected (78 %)	Y	Multiple
Widdowson et al. [27]	Neonatal medium care unit (15), and pediatric and maternity wards at a general hospital; The Netherlands	Closure 1: no new admissions and discharge of all cases	Wave 1: 2 Wave 2: 5 End of outbreak: 2	56/358 in total	Y: relapse after 2w	Multiple
		Closure 2: no admissions and emptied of all patients (2 closures: 3d, 7d)				
*Salmonella panama*						
Kienitz et al. [24]	Pediatric ward in a specialty hospital; Germany	No new admissions	3	16 in total	N: new cases after closure	NA
Small round structured virus						
Green et al. [29]	Wards and a day hospital at a mentally infirm hospital; England	No new admissions; no transfers (17d)	5	13/21 in total	N: new cases after measures	NA
Small round structured virus and small round featureless virus						
Cunney et al. [33]	Geriatric, general, and neighboring wards; Ireland	No new admissions; limited transfers (15d)	5	47 in total: 1 (2 %) died	Y	All

*d* days, *w* weeks, *m* months, *y* years

^a^Includes deaths directly, indirectly, and attributable to infection

^b^Multiple includes ward closure

We identified 17 studies [21–37] on outbreaks involving *C. difficile*, norovirus, rotavirus, *Salmonella panama*, small round structured virus, or small round structured virus and small round featureless virus. The primary mode of transmission for all these pathogens is direct person-to-person contact and indirect contact with contaminated surfaces [20]. The outbreaks occurred at single facilities, of which four occurred at the facility-wide level, and resulted in gastrointestinal system colonization and/or infection among 3–116 inpatients. Between two and ten intervention strategies were used in conjunction with ward closure to control the outbreaks.

The definition of ward closure varied across the studies, and ward closure lasted between 3 days and 1 month among the studies that reported length of closure. Six studies defined ward closure as prohibiting new admissions to the affected clinical area (i.e., unit/ward/bay) [21–26]. Widdowson and colleagues reported on a study that utilized a phased approach, first halting new admissions and discharging all cases, then halting all admissions and discharging all patients from the area [27]. Three studies described completely stopping both admissions and transfers [28–30]. New admissions and transfers were stopped and transfers were limited in the studies by McCall and Smithson and by Stevenson and colleagues [31, 32]. In addition to stopping new admissions, transfers were limited in two studies [33, 34] and discharges were limited in one study [35]. In Hoffman and colleagues’ study, only transfers were limited [36]. One study did not specifically describe their definition of closure [37].

Of nine studies that reported achieving outbreak containment, six attributed it to all the measures used [21, 25, 26, 28, 31, 33], two studies attributed it to multiple, but not all the measures used [27, 37], and one study did not specify which measures contributed to the outcome [36]. In two studies, the reduced number of new cases of colonization and infection was attributed to all the measures used [30, 32]. In two other studies, the authors were uncertain which measures contributed to the reduced number of new cases in one [35], while authors of the other did not report which measures contributed to the reduced number of new cases [29]. Kienitz and colleagues reported that new cases continued to be identified until the pediatric ward was closed; however, newly admitted patients became infected until commercial milk was found to be the source of the outbreak [24]. In three studies, the authors did not report whether the outbreaks were controlled; however, they reported that either all or a number of the measures that were used could be effective at achieving outbreak control [22, 23, 34].

### Respiratory system (Table 2)

**Table 2:** Summary table for accepted studies—respiratory system

Study	Setting (beds); country	Definition of ward closure (length)	Other measures	Inpatient outcomes (includes index case(s))^a^	Controlled (Y, N, NA)	Due to^b^
Influenza A						
Horcajada et al. [40]	Infectious disease and AIDS wards (23) at a tertiary care hospital (800); Spain	No new admissions (2w)	7	8/23 in total	Y	All
Risa et al. [43]	Adult behavioral health unit (26) at a veterans hospital; USA	No new admissions	9	8/26 in total	Y	Multiple
Sartor et al. [44]	Internal medicine unit (19) at a medical school affiliate (700); France	No new admissions	3	9/22 in total: 2 (22 %) positive	Y	NA
Wong et al. [47]	General medical ward, 3 bays (30); Hong Kong, China	No new admissions; no transfers (8d)	5	9/60 in total	Y	All
Parainfluenza						
Moisiuk et al. [42]	Tertiary obstetric-neonatal facility (20); Canada	No new admissions (3w)	8	12/19 in total:	Y	Hand hygiene
				6 (50 %) positive		
Parainfluenza and respiratory syncytial virus						
Jalal et al. [41]	Adult hematology unit (58) at a teaching hospital; UK	No new admissions (2m)	5	30 in total (19 PIV-3, 7 RSV, 4 with both): 11 (37 %) died	Y	Multiple
Severe acute respiratory syndrome						
Gopalakrishna et al. [46]	3 tertiary hospitals (1400, 1600, unknown); Singapore	Hospital 1: hospital-wide undefined closure	Hospital 1: 7	Hospital 1: 11 in total	Y (all 3 hospitals)	All (all 3 hospitals)
		Hospital 2: no new admissions and discharges	Hospital 2: 4	Hospital 2: 12 in total		
		Hospital 3: no new admissions and discharges (10d)	Hospital 3: 1	Hospital 3: 6 in total		
Liu et al. [48]	Primary and tertiary care at a referral medical center (2300); Taiwan, China	Undefined closure	12	16 in total: 4 (25 %) died	Y	All
Owolabi and Kwolek [38]	Obstetrical unit at a general hospital; Canada	SARS 1: limited new admissions	SARS 1 (27d): 8	NA	Y	NA
		SARS 2: no new admissions (45d)	Days 5–49: 3			
*Streptococcus pneumoniae*						
Subramanian et al. [45]	ENT ward at a teaching hospital; UK	No new admissions; no discharges (1w)	4	7 in total	Y	NA
*Streptococcus pneumoniae and Streptococcus*						
Denton et al. [39]	Adult oncology unit (34); UK	No new admissions (11d)	5	8 in total	Y	Closure

*d* days, *w* weeks, *m* months, *y* years

^a^Includes deaths directly, indirectly, and attributable to infection

^b^Multiple includes ward closure

Eleven studies examined outbreaks of influenza A, parainfluenza, parainfluenza and respiratory syncytial virus, severe acute respiratory syndrome, *Streptococcus pneumoniae*, or *Streptococcus pneumoniae* and *Streptococcus* spp. [38–48] The primary mode of transmission for these pathogens is via the combination of both droplet through respiratory secretions and direct and indirect contact [20]. The outbreaks occurred in one to multiple wards/units at single facilities, of which two were at the facility-wide level, and one was at multiple hospitals. The outbreaks resulted in respiratory system infection and/or colonization among 7–30 inpatients; the number of affected patients was not reported in one study [38]. In addition to ward closure, one to nine other interventions were used to control the outbreaks.

The affected clinical area was closed to new admissions in six studies [39–44]. New admissions were stopped in addition to discharges in two studies [45, 46] and transfers in another [47]. Liu and colleagues reported that construction work was undertaken during closure; however, they did not specify the details and length of closure [48]. In the study reported by Owolabi and Kwolek, admissions were initially limited then completely stopped [38]. Ward closure lasted from 1 week to 2 months in six studies; the length was not clear in two studies.

In the studies that achieved outbreak containment, this outcome was attributed to all the measures used in four studies [40, 46–48], and multiple, but not all the measures used in two others [41, 43]. Outbreak containment was attributed specifically to closure in one study [39] and hand hygiene in another [42]. In three studies, the authors did not report which measures contributed to outbreak control [38, 44, 45].

### Other and multiple/mixed systems: predominant colonization (Table 3)

**Table 3:** Summary table for accepted studies—other and multiple/mixed systems with predominant colonization

Study	Setting (beds); country	Definition of ward closure (length)	Other measures	Inpatient outcomes (includes index case(s))^a^	Controlled (Y, N, NA)	Due to^b^
*Enterococcus*						
Delamare et al. [49]	Adult ICU (16); France	Limited transfer; 4 ICU beds closed (8w)	4	15 in total	Y	Multiple
Iosifidis et al. [53]	Pediatric oncology department (16) at a teaching hospital; Greece	No new admissions (3m)	9	21/32 in total: 1 (5 %) died	Y	All
van der Steen et al. [54]	Internal medicine/nephrology and dialysis ward; The Netherlands	No new admissions (12d)	7	12/91 in total: all positive	Y	All
*Escherichia coli*						
Giuffrè et al. [52]	NICU (16) at a teaching hospital; Italy	No new admissions (3m)	4	15/103 in total	Y	All
van der Zwet et al. [55]	Surgical ward in a specialty hospital; The Netherlands	No new admissions (~3d)	5	8 in total	N: 3 patients colonized after	NA
*Klebsiella pneumoniae*						
Rettedal et al. [50]	NICU (21) at a teaching hospital; Norway	No new admissions; limited transfers (70d)	11	59 in total: 1 (2 %) infection	Y	All
*Staphylococcus aureus*						
Barrett [51]	2 adjacent orthopedic wards; England	Unspecified closure	6	15 in total: all positive	Y	Antibiotic treatment
Troelstra et al.	A military hospital; The Netherlands	No new admissions (29d)	4	3 in total	Y	Environmental disinfection

*d* days, *w* weeks, *m* months, *y* years

^a^Includes deaths directly, indirectly, and attributable to infection

^b^Multiple includes ward closure

The mode of transmission for the microbes described within this category is via contact [20]. Eight studies reported on outbreaks of *Enterococcus*, *Escherichia coli*, *Klebsiella pneumoniae*, or *Staphylococcus aureus* that resulted predominantly in colonization and involved 3–59 patients at single facilities [49–56]. Between 4 and 11 other interventions were used in addition to ward closure to control the outbreaks.

In six studies, no new admissions were permitted to the affected clinical area. Ward closure entailed limiting transfers and partial closure of four beds in the outbreak described by Delmare and colleagues [49] and restricting admissions and limiting transfers in the outbreak described by Rettedal and colleagues [50]. The length of closure ranged from approximately 3 days to 3 months. Barrett and colleagues did not specify their use of closure [51].

The authors of four studies attributed outbreak containment to all the measures implemented [50, 52–54]. Delamare and colleagues attributed control to multiple measures [49]. Barrett and colleagues attributed control to treating nasal carriers with nasal mupirocin [51], and van der Zwet and colleagues attributed control to cohorting of colonized patients [55]. Additional patients became colonized after control measures were implemented in one study [55].

### Other and multiple/mixed systems: predominant infection (Table 4)

**Table 4:** Summary table for accepted studies—other and multiple/mixed systems with predominant infection

Study	Setting (beds); country	Definition of ward closure (length)	Other measures	Inpatient outcomes (includes index case(s))^a^	Controlled (Y, N, NA)	Due to^b^
*Acinetobacter baumannii*						
Zanetti et al. [62]	Burn ICU (7); Switzerland	No new admissions (Phase 2: 2.5 m)	Phase 1: 4	5 in total (Phase 1: 2/3; Phase 2: 6/9)	Y	NA
			Phase 2: 3			
Adenovirus						
Finn et al. [58]	Intensive (16) and intermediate (18) care at a teaching hospital; USA	No new admission (ICN: 19d; MCN: 2w)	8	9/34 in total (2, 7): 3 (33 %) positive, 2 (22 %) died	Y	All
Fujiwara et al. [65]	Ophthalmology ward at a teaching hospital; Japan	Limited then no new admissions (16d)	5	17 in total	Y	All
Hamada et al. [67]	Ophthalmology unit at a teaching hospital; Japan	Closure undefined	5	18 in total	Y	All
Kaneko et al. [64]	Ophthalmology ward at a teaching hospital; Japan	Limited admissions (1m)	8	47 in total	Y	Environmental disinfection
Echo 19 virus						
Purdham et al. [61]	Neonatal unit; England	No new admissions (9d)	6	12 in total: 1 (8 %) died	Y	NA
*Enterobacter cloacae*						
Dalben et al. [57]	Neonatal unit (63) at a teaching hospital (2200); Brazil	No new admissions	5	7 in total: 4 (57 %) died	Y	Multiple, excluding closure
*Escherichia coli*						
Lahoucine et al. [66]	Adult, pediatric hematology, oncology ward (36); Morocco	Closure undefined (1w)	1	6 in total: 5 (83 %) died	Y	NA
*Klebsiella pneumoniae*						
Moodley et al. [60]	Intensive care/high care area (34) at a regional hospital; USA	No new admissions	4	26 in total: 22 (85 %) died	Y: after other measures	Multiple, excluding closure
*Pseudomonas aeruginosa*						
Gupta et al. [59]	NICU; India	No new admissions	5	48/2177 in total over 6 outbreaks: 11 (23 %) died	N: reduced cases	All
Zawacki et al. [63]	NICU (18) at a pediatric hospital; USA	No new admissions	11	4 in total: 2 (50 %) died	Y	Treating HCW carriage
*Staphylococcus aureus*						
Noone and Griffiths [7]	Gynecological, neurosurgical, gastroenterological, 2 acute general surgical wards; England	No new admissions	4	28 in total (25 prior to cleaning ward, 3 after cleaning)	Y	Do not know

*d* days, *w* weeks, *m* months, *y* years

^a^Includes deaths directly, indirectly, and attributable to infection

^b^Multiple includes ward closure

The major mode of transmission for the microbes described within this category is via contact with the exception of adenovirus and the echovirus where both contact and droplet transmission occur [20]. We identified 12 studies that reported on outbreaks of *Acinetobacter baumannii*, adenovirus, echo 19 virus, *Enterobacter cloacae*, *Escherichia coli*, *Klebsiella pneumoniae*, *Pseudomonas aeruginosa*, or *Staphylococcus aureus* that resulted predominantly in infection among 4–48 patients [7, 57–67]. The authors reported using 1–11 interventions in addition to ward closure.

Ward closure involved closing the affected clinical area to new admissions in eight studies [7, 57–63]. Admissions to the affected clinical area were limited in two studies [64]. In the study reported by Fujiwara and colleagues [65], admissions were first limited and then completely stopped. Two studies did not define the closure used during the outbreak [66, 67]. Ward closure lasted from 1 to 10 weeks in seven studies that reported the length of closure.

Authors reported achieving outbreak control in 11 studies. This outcome was attributed to all the measures used in three studies [57, 65, 67]. Successful containment was attributed to multiple measures, excluding ward closure, in two studies [57, 60] and the treatment of HCW carriers in another [63]. How outbreak containment was achieved was unknown in three studies [7, 61, 62]. Gupta and colleagues attributed the reduction of new cases to all measures instituted [59]. Kaneko and colleagues attributed control specifically to environmental disinfection [64]. One study did not identify which measure(s) contributed to control [66].

### Other and multiple/mixed systems: combination of colonization and infection (Table 5)

**Table 5:** Summary table for accepted studies—other and multiple/mixed systems with combination of colonization and infection

Study	Setting (beds); country	Definition of ward closure (length)	Other measures	Inpatient outcomes (includes index case(s))^a^	Controlled (Y, N, NA)	Due to^b^
*Acinetobacter baumannii*						
Alfandari et al. [97]	ICU (16) and infectious diseases unit at a general hospital (400); France	Second outbreak: no new admissions	Stage 1: 8 Stage 2: 2	20 in total: 15 infected (75 %), 6 died (30 %)	Y	Multiple, particularly equipment disinfection
Ayraud-Thévenot et al. [69]	Surgical (15), medical (12), and intermediate care units (6) at a teaching hospital (1500); France	First outbreak: undefined partial and complete closure (1 m) Second outbreak: no new admissions	First outbreak: 7 Second outbreak: 3	First outbreak: 20 in total: 16 (80 %) asymptomatic, 4 (20 %) infected, 1 (5 %) died Second outbreak: 7 in total: 3 (43 %) asymptomatic, 4 (57 %) infected	Y	Closure
Enoch et al. [102]	Neurosciences critical care unit (21) and general ICU (14) at a teaching hospital (1100); UK	Phase 2: limited transfers (16d)	Phase 1: 5	19 in total (16, 3): 8 (42 %) died; 9 (47 %) positive; 10 (53 %) infected	Y	All
			Phase 2: 6			
			Phase 3: 5			
Koeleman et al. [76]	Surgical ward at a teaching hospital; The Netherlands	Stage 3: no new admissions (12d)	Stage 1: 2	13 in total: 8 (62 %) infected, 5 (38 %) colonized	Y	Closure for disinfection
			Stage 2: 3			
			Stage 3: 2			
Landelle et al. [77]	5 ICUs: 4 surgical and 1 medical (95 total) at a teaching hospital (860); France	No new admissions	Phase 1: 5	86 in total	Y	Multiple excluding closure
			Phase 2: 2			
			Phase 3: 3			
			Phase 4: 1			
			Phase 5: 4			
Simor et al. [92]	Burn unit (14) at a teaching hospital; Canada	No new admissions (1w)	8	31/247 in total: 18 (58 %) infected; 7 (23 %) died	Y	Multiple
Wagenvoort et al. [100]	ICU in a specialty hospital; The Netherlands	Limited admissions	3	66 in total	Y	All
Coxsackie virus						
Konjajev et al. [110]	Neonatal unit, Yugoslavia	Unspecified closure	2	6 in total	Y	Closure
*Enterobacter aerogenes*						
Piagnerelli et al. [109]	Geriatric acute unit (30); Belgium	Unspecified (20d)	4	12 in total	Y	Closure
*Enterococcus faecium*						
Bartley et al. [70]	Renal unit (30), infectious diseases unit at a teaching hospital (800); Australia	No new admissions (pre- and during outbreak)	Prior to outbreak: 5	47 in total	Y	Multiple
			Outbreak: 11			
Ergaz et al. [73]	NICU (16); Israel	No new admissions (1m)	6	11/18 in total: 3 (27 %) infections; 8 (73 %) positive	Y	Multiple
Liu et al. [80]	Surgical (15) and emergency (10) ICUs at a teaching hospital (1500); China	No new admission area (2w)	5	8 in total	Y	All
Moretti et al. [83]	Gastroenterology clinic and several units at a teaching hospital; Brazil	No new admissions (15d)	Phase 1: 5	321 in total: 16 (5 %) infected	N: significant (*p* < 0.001) reduction in cases	Multiple
			Phase 2: 3			
Sample et al. [95]	Hematology–oncology unit (32) at a teaching hospital (1100); Canada	Stage 1: limited admissions	5	16 in total: 3 (23 %) died	Y	All
		Stage 2: no new admissions				
*Enterobacter cloacae*						
Donkers et al. [72]	NICU at a teaching hospital; Holland	No new admissions (<1m)	5	26 in total: 2 (8 %) died	Y	Dedicated and disposable equipment
Modi et al. [81]	NICU at a maternity hospital; England	No new admissions	3	12 in total: 6 (50 %) positive; 6 (50 %); 2 (17 %) died	Y	All
van den Berg et al. [94]	NICU(15) at a tertiary hospital (950); The Netherlands	No new admissions	Stage 1: 5	32 in total: 2 (6 %) infected	Y	Mainly equipment disinfection
			Stage 2: 5			
*Escherichia coli*						
Moissenet et al. [82]	Neonatal ward (30) at a children’s teaching hospital; France	Phase 2: no new admissions (1w ≥ 2w) Phase 3: no new admissions (1w ≥ 2w)	Phase 1: 5	26/59 in total	Y	Ward closure
			Phase 2: 3			
			Phase 4: 4			
Quinet et al. [85]	Neonatal unit (30); France	Limiting admissions to infants born at the hospital; no new admissions (6w)	6	27/59 neonatal patients affected	Y	Closure
*Klebsiella pneumoniae*						
Carbonne et al. [101]	Seven hospitals; France	All 7 hospitals: limited transfers	7	13 in total: 4 (31 %) infected; 9 (69 %) positive	Y	All
Grogan et al. [103]	Pediatric intensive care; Ireland	No new admissions; limited discharges (1w)	10	3 in total	Y	Multiple
Kassis-Chikhani et al. [104]	Abdominal surgery care center (81) in a teaching hospital (716); France	Limited new admissions; limited transfers	First 7m: 5	8 in total (6, 2); 4 (50 %) died	Y	Multiple
			Next 4.5m: 6			
Laurent et al. [105]	4 ICUs (6, 6, 8, 1) at a teaching hospital (858); Belgium	Limited transfers, no new admissions	11	30 in total: 9 (30 %) infected; 3 (10 %) died	Y	All
Macrae et al. [106]	Intensive care section (8) and special care section (15) at a neonatal unit; UK	Stage 1: limited transfers (10d) Stage 2: no new admissions (39d)	Stage 1: 5 Stage 2: 11	22 in total: 15 (68 %) positive; 1 (14 %) died	Y	Temporary ward opened so infected ward could be closed for disinfection
McKee Jr. et al. [75]	Intensive care nursery (30) at a teaching children’s hospital; USA	No new admissions (2w)	6	26/232 in total: 21 (81 %) positive; 5 (19 %) infected; 1 (4 %) died	Y	All
Reish et al. [89]	NICU at a tertiary care center; Israel	No new admissions	3	8 in total: 5 (63 %) infected; 3 (37 %) positive; 3 (37 %) died	Y	All
Ritter et al. [90]	Surgical ward in a specialty hospital; The Netherlands	No new admission	4	11 (10 %) infected; 4 (36 %) died	Y	Disinfection during closure
Parvovirus						
Pillay et al. [99]	General pediatric ward; England	Limited admissions	5	9 in total: 2 (22 %) patients infected	Y	All
Seng et al. [91]	Adult surgical unit (28); England	No new admissions	3	3/6 in total: 3 (50 %) positive; 3 (50 %) infected	Y	Author does not know
*Salmonella*						
Newman [98]	NICU (18) at a teaching hospital; Ghana	Limited admissions	3	21/72 in total	Y	Aseptic measures and closure
*Serratia marcescens*						
Assadian et al. [68]	NICU (8) at a teaching hospital (2168); Austria	No new admissions (10d)	First outbreak: 4	8 in total: 5 (63 %) infected; 3 (37 %) positive	N: 2 of different isolates after 41d	NA
			Second outbreak: 2			
Lewis et al. [78]	Neonatal; England	No new admissions (7w)	9	13/24 in total: 2 (15 %) died	Y	Closure
Maragakis et al. [79]	NICU (36) at a tertiary care hospital (926); USA	No new admissions	9	18 in total	Y	Closing beds to enable cohorting
*Staphylococcus aureus*						
Boyce et al. [107]	Burn unit (4) at a teaching hospital (580); USA	Stage 1: restricted admissions (3 occasions)	5	245 in total: 151 infections; 40 (26 %) deaths	N: new cases until permanent closure	Permanent closure
		Stage 2: permanently closed				
Danchivijitr et al. [96]	Burn unit; Thailand	Phase 1: No new admission (2m)	Phase 1: 3	19/29 in total: 14 (74 %) infected; 5 (26 %) positive; 5 (26 %) died	N	NA
			Phase 2: 2			
Hill and Ferguson [74]	Special baby care unit (24) at a university hospital; UK	Stage 1: no new admissions (2 occasions: 10d, 2w)	9	35/315 in total: 2 (6 %) infected; 1 (3 %) died	N	NA
Kluytmans et al. [108]	Hematology unit and surgical unit at a teaching hospital; The Netherlands	Undefined closure	Outbreak 1: 5	27 in total: 24 (89 %) infected; 5 (19 %) died	Y	Mainly external cohort isolation
			Outbreak 2: 4			
Price et al. [84]	Neonatal medical and surgical unit; England	No new admissions	13	11 in total: 2 (18 %) infected; 1 (9 %) died	Y	All
Rampling et al. [87]	Male surgical (37) and female surgical (32) wards; UK	Closure of one bay at a time; no new admissions	Phase 1: 7	69 in total (66, 3)	Y	Closure and environmental disinfection
			Phase 2: 5			
Rashid et al. [88]	Burn unit (12) at a regional hospital; Ireland	No new admissions (2w)	7	18/ 176 in total: 3 (17 %) infected	Y	All
Teare et al. [93]	Burn unit (20) and plastics unit (84); England	No new admission	Stage 1: 1	19 in total	Y	Treatment for HCW
			Stage 2: 2			
			Stage 3: 3			
			Stage 4: 5			
*Streptococcus*						
Deutscher et al. [71]	Long-term acute care hospital; USA	No new admissions (26d)	9	19 in total: 8 (42 %) positive; 3 (16 %) assumed; 8 (42 %) asymptomatic; 2 (15 %) died	Y	All
Ramage et al. [86]	Medical unit (24) at a community hospital (235); Canada	No new admissions	6	3/25 in total: 3 (100 %) died	Y	HCW treatment and infected inpatient deaths

*d* days, *w* weeks, *m* months, *y* years

^a^Includes deaths directly, indirectly, and attributable to infection

^b^Multiple includes ward closure

Of the remaining studies, 43 reported outbreaks that affected other or multiple organ systems and resulted in both of infection and colonization [68–110]. The major mode of transmission for the microbes described within this category is via contact with the exception of Coxsackie virus and parvovirus where both contact and droplet transmission occur [20]. The studies reported on outbreaks of the following: *Acinetobacter baumannii*, Coxsackie virus, *Enterobacter aerogenes*, *Enterobacter cloacae*, *Enterococcus faecium*, *Escherichia coli*, *Klebsiella pneumoniae*, parvovirus, *Salmonella*, *Serratia marcescens*, *Staphylococcus aureus*, or *Streptococcus*. For all but one study that involved a total of seven hospitals, the outbreaks occurred at one facility and affected a total of 3–245 patients.

Among these studies, the definition of ward closure varied widely and lasted from 1 week to 2 months in 19 studies that reported the length of closure. Ward closure was defined as limiting and then not accepting new admissions to the affected clinical area in 30 studies [68–97], limiting admissions in three studies [98–100] and limiting transfers in two studies [101, 102]. Ward closure entailed both stopping new admissions to the affected clinical area(s) and limiting transfers or discharges in four studies [103–106]. Boyce and colleagues reported that permanent closure of a burn unit was necessary to control a MRSA (methicillin-resistant *Staphylococcus aureus*) outbreak that could not be controlled by the use of other measures, including temporary closure on three occasions [107]. Three studies did not provide specifics of their use of ward closure [108–110].

Successful outbreak containment was reported in the vast majority of the studies. This outcome was attributed to multiple measures in five of the studies [70, 73, 92, 103, 104], multiple measures, excluding ward closure in one study [77], and to all the measures used in 13 of the studies [71, 75, 80, 81, 84, 88, 89, 95, 99–102, 105]. Other studies attributed outbreak control specifically to the closure of the affected ward(s) [69, 78, 82, 85, 109, 110], provision of dedicated and disposable equipment [72], disinfection of equipment [94, 97], construction of a cohort isolation ward outside of the affected hospital [108], disinfection of the affected clinical area(s) during closure [76, 87, 90, 98, 106], cohorting enabled by ward closure [79], and treatment of healthcare workers for carriage [93], as well as death of the infected inpatients [86]. Seng and colleagues reported not knowing which measure(s) contributed to outbreak containment [91]. While the authors of three studies reported unsuccessful containment [68, 74, 96]. Boyce and colleagues reported that permanent closure of the burn unit, the source unit, was necessary to control a MRSA outbreak on other units [107]. Moretti and colleagues reported that a combination of measures contributed to a statistically significant reduction (*p* < 0.001) in the number of cases of colonization and infection [83].

### Studies on infection prevention and control policies or specific interventions (Table 6)

**Table 6:** Summary table for accepted studies—infection prevention and control policies and specific interventions

	Setting (beds); country	Study length	Definition of ward closure	Main interventions	Outcomes
Gastrointestinal: norovirus					
Haill et al. [13]	Teaching hospital (1200); England	2005–2011	Unspecified closure	2005–2007: ward closure; meet criteria before reopening; terminal cleaning	Many norovirus outbreaks can be controlled by bay closures when combined with adequate infection control support
				2007–2011: isolation and cohorting in bays to facilitate disinfection	New policy led to reduction in: duration of closure from 6d to 5d and bed-days lost from 180 to 96
Illingworth et al. [12]	Teaching hospital (1100); England	2006–2010	Unspecified bay closures	2006–2008: Early ward closure	New policy led to significant reduction in: length of closure (*p* < 0 .041) and in bed-days lost (*p* < 0.001)
				2008–2010: Closure of ward bays; architectural installation; environmental disinfections; enlarged infection control team	
Other and multiple/mixed systems with predominant infection *Acinetobacter baumannii*					
García et al., 2009 [114]	2 ICUs (30, 24) at a tertiary hospital (934); Spain	2006–2007	Unspecified sequential closure	Cleaning/disinfection (intervention); clinical procedures limited; isolation; dedicated HCW; contact precautions; HCW and environmental screening; education	Cleaning/disinfection led to a decrease from 3.2 to 1.6 episodes per 100 patients, and incidence density of 9.2 to 5 infections per 1000d of stay
Other and multiple/mixed systems with combination of colonization and infection: *Staphylococcus aureus*					
Farrington et al. [111]	Teaching hospital (1000); England	1985–1997	No new admissions; limited transfers	1985–1994: MRSA screening upon admission to ICU; isolation; ward closure; disinfection	Relaxation of policy and increase MRSA upon admission led to an increased in MRSA cases from 1 to 2 in 1994 to 74 cases in 1997
				1994–1997: relaxed closure/reopen and screening criteria	
Selkon et al. [112]	General hospital (1000 beds); England	1967–1978	Unspecified closure	1967–1972: ward closure; standard barrier nursing methods	Ward closure and barrier nursing did not control the outbreaks
				1972–1978: limited transfer; construction of a isolation unit with control ventilation	New policy led to reduction in incidence rate of MRSA infection from 6.57 to 5.08 cases per 1000 admissions; from 130 to 14 cases of infection
Combination of colonization and infection: *Clostridium difficile and Staphylococcus aureus*					
Stone et al. [113]	Acute medical wards (66) at an acute geriatrics hospital; England	1994–1996	Unspecified closure	1994–1995: ward closure; national guidelines	Ward closure and national guidelines did not control the outbreaks
				1995–1996: hand hygiene; education/ communication; antimicrobial treatment restricted	New policy led to reduction in: incidence rate of *C. difficile* infection from 3.35 cases to 1.94 cases per 100 admissions (*p* < 0.05), and MRSA incidence from 3.95 to 194 cases per 100 admissions (*p <* 0.01)

*d* days, *w* weeks, *m* months, *y* years

We identified six studies that focused on the impact of specific infection prevention and control policies or a control intervention [12, 13, 111–114]. The mode of transmission for the microbes described within this category is via contact [20]. All the studies involved new policies and/or interventions that influenced ward closure prerequisites, ward re-opening criteria, and impact of alternate measures to that of ward closure on outbreak control. Recorded outcomes of the new policies and interventions include duration of closure in two studies [12, 13], bed-days lost in two studies [12, 13], and rate of new infection cases in four studies [111–114].

In two studies reporting on norovirus outbreak(s), bay closures supplemented with other measures were reported to have a greater impact on the reduction of closure length and bed-days lost than ward closure as a primary intervention [12, 13]. Although a number of other interventions were used, Garcia and colleagues attributed a reduction in the episodes and incidence density of infections to cleaning and disinfection during sequential closure of affected clinical areas [114]. In two other studies, the authors indicated that successful containment could not be achieved when ward closure was used as part of the control strategy. In their 11-year study, Selkon and colleagues found that a dedicated isolation unit with controlled ventilation was crucial to reducing the incidence rate of nosocomial MRSA infections [112]. Stone and colleagues observed a significant decrease in the incidence rates of *C. difficile* infection and MRSA when a new policy entailing hand hygiene, education, and restriction on antimicrobial treatment was implemented [113]. Lastly, Farrington and colleagues reported on the incidence of MRSA during the application of a MRSA control policy aimed at eradication over 10.5 years and relaxation of the same policy for the next 1.5 years [111]. The authors reported a notable increase in MRSA incidence following the relaxation period; however, the authors noted that the increase could not be solely attributed to the relaxation of the policy as there was also an increase in admission of MRSA carriers.

### Risk of bias

Owing to the nature of the studies included in this review, a number of potential confounders and sources of bias were identified. Firstly, none of the studies controlled for confounding, and the majority of them did not address the confounding factor bias when discussing the impact of the interventions used. All of the studies used ward closure in combination with other interventions, and as such, the impact of each measure on outbreak containment could not be determined. Relatedly, there may also have been a potential for a dose–response relationship in studies that increased the extent of ward closure, for example, from closing the unit to select admissions to closing to all admissions. Secondly, there is the potential for selection bias in studies that did not use epidemiological typing and, subsequently, could not confirm that all affected patients were colonized or infected with the same strain of the causative pathogen. Another source of bias stems from the selection of specific outcomes. Furthermore, some of the case definitions relied on the presence of symptoms and did not confirm cases with any diagnostic method or epidemiological typing, resulting in case finding bias. The studies could have also been subject to recall bias as the vast majority of articles are retrospective. As all the articles included in this study reported on successfully controlled outbreaks, it is highly likely that the reviewed literature is vulnerable to publication bias. Many of the articles failed to address these potential sources of bias that may have contributed to the main findings and, particularly, the impact of ward closure on containing the outbreaks. This failure may be attributed to the retrospective and observational nature of outbreak investigation studies. Fourthly, definitions of ward closure were varied between studies, potentially creating bias in understanding the impact of ward closure and in determining whether the studies used complete or partial closure.

## Discussion

We sought to identify studies that describe the use of ward closure as an intervention in outbreak control and determine its importance. Our systematic review expands on existing work by providing an extensive review of the epidemiological literature on the use of ward closure as an intervention to control outbreaks of pathogenic microbes among inpatients hospitalized in acute care settings. We identified 97 studies that described the use of ward closure as part of a bundled approach to their strategy. None of the studies used ward closure in a setting where it was able to be isolated as a singular control measure, limiting our assessment of the direct efficacy of ward closure on outbreak containment, which was one of our primary objectives.

It was not possible to draw any firm conclusions about the impact or effect of ward closure from the studies for a number of reasons. Firstly, the use of “ward closure” varied considerably within the papers included in the review. Our review was unable to identify whether partial or complete closure was instituted in the vast majority of the studies, as precise definitions were not used to describe the type or nature of ward closure. The results suggest that there is not a universal definition of “ward closure”; rather, ward closure refers to restrictions on patient movement into and out of a unit/ward or a facility and could encompass a number of qualities and multiple phases and/or degrees of application. Secondly, with the exception of the prevention and control policy and intervention studies, all of the studies of the included papers were reports or descriptions of outbreak investigations. As investigators could not manipulate exposures (i.e., the outbreak), all outbreak studies were observational in nature and the results were thus susceptible to a number of potential confounders. The vast majority of the included articles did not record these potential confounders or were not adjusted accordingly in any type of additional analysis. The studies were vulnerable to multiple biases, including confounding factor bias, publication bias, and recall bias, and none of them reported taking measures to prevent them or address their source. As Cooper and colleagues [115] noted, these studies generally did not meet standards of planned research as most, if not all, outbreak reports were written retrospectively. Thus, the majority of the included studies were considered to be of poor quality as the nature of outbreak investigation reports rendered the use of high-quality study designs such as randomized controlled trials unfeasible. Thirdly, all of the studies used combinations of measures in an attempt to reduce or terminate transmission. As a result, the relative contribution of each measure, and especially ward closure, could not be determined. The lack of attribution could be due to the reporting style, as many authors listed all the measures used without providing information on whether they were instituted consecutively or concurrently. Overall, ward closure was generally used at a late stage in conjunction with other measures, primarily hygienic and disinfection measures. Finally, considerable variability across the studies limits the generalizability and comparability of the outcomes of the studies. Thus, we considered the conclusions to be very weak when authors stated that the containment of an outbreak could be attributed to any one of the measures used as potential alternative factors accounting for the main findings could not be dismissed.

Our review highlights potential areas for further research on the role of ward closure as an intervention measure for managing and terminating outbreaks. Improving the quality of reporting can be a first step to addressing the difficulties in assessing the applicability and generalizability of these studies [116]. Given the complexities of outbreak investigations and the nature of the studies, clear and detailed reporting enables greater understanding of the context of the outbreak, the outbreak itself, and the control measures used, which may or may not include ward closure. Reports of outbreaks that use ward closure should include a clear definition or description of ward closure, timing of ward closure, and at which point it was used in the investigation. Given the nature of outbreak investigations, an experimental design would not be feasible. However, since the role if any of ward closure in containing outbreaks is unknown, quasi-experimental design is ethically unacceptable. Future research can improve the rigor and internal validity by using study designs of higher quality such as prospective cohort studies and cluster randomized trials. For example, a cluster randomized design study of ward closure, or no ward closure plus a defined bundle of other interventions for specified outbreak organisms, could be conducted.

Further, formal assessment of the frequency and outcomes of unit closure versus no unit closure during an outbreak could be undertaken. This should include gathering information on the type of outbreak where a unit is closed, duration of the outbreak, whether or not the unit is closed, and the impact on patient flow, examining both admission and discharge. While there are some inconsistencies in the quality and format of reporting, there are some metrics that are consistently reported, including number of beds, length of closure, and bed-days lost. This information could inform an economic study using modeling to predict the cost of implementing ward closure. Finally, there are potential ethical and legal considerations in deciding whether to implement closure of care settings during outbreaks that are not addressed in the literature reviewed nor within this review. On the one hand, failure to restrict admissions implies that new and unaffected patients are knowingly admitted to an area known to have ongoing transmission of a potential pathogen; on the other hand, closure of a clinical area may reduce access to care.

While this review was undertaken with rigor and in accordance with the requirements of systematic review methodology, it is important to note its limitations. Firstly, for the majority of articles, data were extracted by a single reviewer; however, initial screening was undertaken rigorously by two reviewers, and disagreements were resolved with a third-party adjudicator. Secondly, the literature available for this review could report a positive effect of ward closure, as it is possible that there are many outbreaks that were controlled without using ward closure and were never published. Similarly, outbreaks where interventions failed to control transmission leading to endemic transmission are less likely to be published. For example, it is common for long-term care facilities to use ward (or facility) closure (along with other interventions) to control gastrointestinal and respiratory outbreaks, and these are seldom published. While the outbreaks are generally controlled and the ward (or facility) is re-opened, the key question is whether ward closure is necessary and effective. Lastly, the review is based on the last electronic search which was completed in July 2014, and as such the review may not be entirely up to date.

It can be concluded that ward closure for containment of outbreaks remains an intervention that is not evidence based in the traditional sense; however, this review demonstrates that ward closure is frequently used and was always used as part of a bundled approach, whether as part of a sequence of, or in parallel with, other interventions, and in this sense, is similar to other public health responses. However, it was interesting to observe that in the majority of the studies in this review, ward closure was applied in the late stages of the overall outbreak response rather than as a first measure. In addition, in 16 studies, despite the use of ward closure, additional cases continued to be reported, suggesting that ward closure was not an effective intervention in these settings. Other than general wards, which were not described well, burn units (*n* = 3), geriatric wards (*n* = 3), and neonatal intensive care units (*n* = 2) were reported more than once (Tables 1, 3, 4, 5, and 6). The most frequently recorded mode of transmission was contact with viral gastrointestinal-associated viruses (four norovirus and one small round structured virus) and bacterial (*S. panama*, *C. difficile*, *E. faecium*, *and E. coli*), making up 56 % of the pathogenic species. These pathogens are known for their persistence within environmental niches and relative resistance to commonly used disinfectants.

There are also potential ethical considerations in the closure of wards during outbreaks that are not addressed within the context of the reviewed studies and would need to be taken into consideration by infection control personnel and hospital administrators. Admitting new and unaffected individuals to a hospital ward that is known to have ongoing transmission of a potential pathogen, particularly if associated with a high case fatality rate, warrants careful deliberation. The risk of new transmissions needs to be juxtaposed against the failure to contain the outbreak despite closure, the disruption of care delivery, and lack of access to care for other patients and overloading other care units, particularly emergency departments, where the risks of overcrowding and delayed care present other challenges.

With no published controlled studies associated with a benefit from ward closure, infection control practitioners and hospital administrators will need to continue to balance the competing risks, taking into consideration the nature of the outbreak, the type of pathogen and its virulence, mode of transmission, and the setting in which it occurs and take reasonable steps to protect patients, and since ward closure has been used in the past, it will likely continue to be used as an intervention strategy until better quality evidence is available.

## Conclusions

The present systematic review could not ascertain the impact of ward closure on outbreak containment for any of the included studies based on our primary objective. Ward closure was commonly reported as an intervention during the course of a wide range of outbreaks, and outbreak control was described in most settings with the use of ward closure, usually in the late stages of the outbreak and was always used in parallel or in sequence with other interventions. Our results highlight that there is no universal definition of ward closure, as it has been defined in various and imprecise ways in the included studies. Since the published literature to date consists of uncontrolled observational study designs that were vulnerable to a number of potential confounders and biases, the actual impact of ward closure could not be determined. Our review has identified a number of research gaps and new opportunities for future investigations. In particular, the ability to determine the generalizability and applicability of ward closure as a control intervention could be improved by standardizing outbreak investigation reporting to include information on the use, role, precision of definition, and timing of ward closure.

## Additional file

Additional file 1:
**MEDLINE Search Strategy.** (DOCX 23 kb)

## Abbreviations

AIDS: acquired immunodeficiency syndrome
ARO: antibiotic-resistant organism
CDCP: Centers for Disease Control and Prevention
HAI: healthcare-acquired infections
HCW: healthcare worker
ICN: intensive care nursery
ICU: intensive care unit
MCN: Intermediate care nursery
MRSA: methicillin-resistant *Staphylococcus aureus*
NICU: neonatal intensive care unit
PIV: parainfluenza virus
RSV: respiratory syncytial virus
SARS: severe acute respiratory syndrome
WHO: World Health Organization
