# Supplementary material for: Use of ward closure to control outbreaks among hospitalized patients in acute care settings: a systematic review
Source: Syst Rev. 2015 Nov 7;4:152. doi: 10.1186/s13643-015-0131-2 (PMC4636845; doi:10.1186/s13643-015-0131-2)
Supplement: Additional file 1: — MEDLINE Search Strategy. (DOCX 23 kb) [file 13643_2015_131_MOESM1_ESM.docx]

**Search Strategies**

**MEDLINE Search Strategy**

1. exp Health Facility Closure/

2. (hospital* adj3 (close* or closur* or closing)).tw.

3. (ward* adj3 (close* or closur* or closing)).tw.

4. (unit* adj3 (close* or closur* or closing)).tw.

5. (wing* adj3 (close* or closur* or closing)).tw.

6. (partial* adj2 (close* or closur* or closing)).tw.

7. (bay* adj3 (close* or closur* or closing)).tw.

8. (department* adj3 (close* or closur* or closing)).tw.

9. cohorting.tw.

10. or/1-9

11. exp Infection Control/

12. exp Communicable Disease Control/

13. (infection adj3 control*).tw.

14. exp Disease Outbreaks/

15. outbreak.tw.

16. exp Disease Transmission, Infectious/

17. (cross adj2 transmi*).tw.

18. (pathogen* adj2 spread*).tw.

19. (pathogen* adj3 colonis*).tw.

20. (pathogen* adj3 coloniz*).tw.

21. (microb* adj3 colonis*).tw.

22. (microb* adj3 coloniz*).tw.

23. (mycobacteri* adj3 colonis*).tw.

24. (mycobacteri* adj3 coloniz).tw.

25. exp Cross Infection/

26. exp Viruses/

27. Virus Diseases/

28. rhinovirus.tw.

29. exp gram-negative bacteria/

30. exp Influenza, Human/ or (influenza or flu).tw.

31. exp Methicillin-Resistant Staphylococcus aureus/ or exp Staphylococcal Infections/ or (Staphylococcus aureus or mrsa or mssa).tw. 111

32. (exp Vancomycin Resistance/ and exp Enterococcus/) or (Vancomycin resistant Enterococci or vre).tw.

33. (cre or Carbapenem resistant enterobacteri*).tw.

34. (cro or Carbapenem Resistant Organism*).tw.

35. (cpo or Carbapenemase Producing Organism*).tw.

36. exp Enterobacteriaceae Infections/

37. (ndm-1 or New Delhi metallo-beta-lactamase-1).tw.

38. (esbl or Extended Spectrum Beta Lactamase).tw.

39. Drug Resistance, Microbial/ or exp Drug Resistance, Bacterial/

40. (anti microbial resistan* or antimicrobial resistan* or amr).tw.

41. exp Tuberculosis/ or (tb or tuberculosis).tw.

42. exp Nontuberculous Mycobacteria/ or exp Mycobacterium Infections/

43. exp Clostridium difficile/ or (c difficile or cdf or cdi or Clostridium difficile Associated Disease or cdad or bacillus difficilis).tw.

44. exp Norovirus/ or (norovirus or Norwalk virus).tw.

45. exp Caliciviridae Infections/ or calicivirus.tw.

46. exp Rotavirus Infections/ or rotavirus.tw.

47. exp Klebsiella pneumoniae/ or kpc.tw.

48. exp Coronavirus Infections/ or (Middle East respiratory syndrome or MERS-CoV or EMC 2012).tw.

49. (((anti-biotic or antibiotic) adj2 organism*) or (anti-resistant or antiresistant)).tw.

50. ((healthcare adj2 (associated or acquired)) or hospital acquired infection* or hai).tw.

51. (nosocomial or (nosocomial adj2 pathogen*)).tw.

52. exp Communicable Diseases/

53. exp Diarrhea/ or ((hospital adj3 diarrhea*) or (diarrhea* adj2 ill*)).tw.

54. exp Measles/ or (Subacute Measles Encephalitis or sme).tw.

55. oxa 48.tw.

56. exp Common Cold/

57. common cold.tw.

58. exp Adenoviridae/ or exp Mastadenovirus/ or exp Adenoviruses, Human/ or adenovir*.tw.

59. exp Adenoviridae Infections/

60. exp Severe Acute Respiratory Syndrome/ or (severe acute respiratory syndrome or sars).tw.

61. exp Respiratory Syncytial Viruses/ or exp Respiratory Syncytial Virus Infections/ or (respiratory syncytial virus or rsv).tw.

62. exp Human bocavirus/ or bocavirus.tw.

63. exp Pneumovirus Infections/ or exp Metapneumovirus/ or metapneumovirus.tw.

64. exp Enterovirus Infections/ or exp Enterovirus/ or enterovirus.tw.

65. exp Parvovirus B19, Human/ or Parvoviridae Infections/ or exp Erythema Infectiosum/

66. exp Parechovirus/ or parechovirus.tw.

67. (acute respiratory infection* or acute respiratory tract infection*).tw.

68. exp Orthomyxoviridae Infections/

69. (avian and (influenza or flu)).tw.

70. exp Acinetobacter/ or exp Acinetobacter Infections/ or acinetobacter.tw.

71. HCoV EMC 2012.tw.

72. or/11-72

73. 10 and 72

**Lilacs Search Strategy (Using the VHL Search Portal Interface)- Search run on January 31^st^**

| Searches | Results |
| --- | --- |
| (tw:(facility OR facilities)) AND (tw:(close$ OR closure$)) AND (tw:(outbreak$ OR disease$ transmi$ OR spread$ OR coloniz$ OR colonis$)) AND (instance:"regional") AND ( db:("LILACS")) | 33 |
| (tw:(facility OR facilities)) AND (tw:(close$ OR closure$)) AND (tw:(infection$ OR virus$ OR pathogen$ OR microb$ OR mycobacteri$ OR hai OR nosocomial)) AND (instance:"regional") AND (db:("LILACS")) | 11 |
| (tw:(facility OR facilities)) AND (tw:(close$ OR closure$)) AND (tw:(tuberculosis OR measles OR staphylococc$ OR norovirus OR entero$ OR gastroenteritis)) AND (instance:"regional") AND ( db:("LILACS")) | 7 |
| (tw:(facility OR facilities)) AND (tw:(close$ OR closure$)) AND (tw:(influenza OR flu OR mdn1 OR "midasin homolog" OR esbl OR "Extended Spectrum Beta Lactamase" OR "Clostridium difficile" OR calicvir$ OR Rotavirus OR Klebsiella OR kpc OR Coronavirus OR "Middle East respiratory syndrome" OR diarrhea OR Adenovir$ OR Mastadenovirus OR "severe acute respiratory syndrome" OR sars OR "respiratory syncytial" OR bocavirus OR pneumovirus OR metapneumovirus OR parvovirus OR parechovirus OR orthomyxovirus)) AND (instance:"regional") AND ( db:("LILACS")) | 0 |
| (tw:(facility OR facilities)) AND (tw:((halt$ OR limit$ OR delay$) AND (admit$ or admis$))) AND (instance:"regional") AND ( db:("LILACS")) | 11 |
| (tw:(unit$) AND (close$ OR closure$)) AND (tw:(outbreak$ OR disease$ transmi$ OR spread$ OR coloniz$ or colonis$)) AND (tw:(infection$ OR virus$ OR pathogen$ OR microb$ OR mycobacteri$ OR hai)) AND (instance:"regional") AND ( db:("LILACS")) | 90 |
| (tw:(unit$) AND (close$ OR closure$)) AND (tw:(influenza OR flu OR staphylococc$ OR Klebsiella OR diarrhea OR entero$ OR tuberculosis OR measles OR "respiratory syncytial")) AND (instance:"regional") AND ( db:("LILACS")) | 36 |
| (tw:(unit$) AND (close$ OR closure$)) AND (tw:(mdn1 OR "midasin homolog" OR esbl OR "Extended Spectrum Beta Lactamase" OR "Clostridium difficile" OR norovirus OR calicvir$ OR Rotavirus OR kpc OR Coronavirus OR "Middle East respiratory syndrome" OR Adenovir$ OR Mastadenovirus OR "severe acute respiratory syndrome" OR sars OR bocavirus OR pneumovirus OR parvovirus OR parechovirus OR orthomyxovirus OR gastroenteritis)) AND (instance:"regional") AND ( db:("LILACS")) | 0 |
| (tw:(unit$)) AND (tw:((halt$ OR limit$ OR delay$) AND (admit$ or admis$))) AND (tw:(outbreak$ OR disease$ transmi$ OR spread$ OR coloniz$ OR colonis$)) AND (instance:"regional") AND ( db:("LILACS")) | 57 |
| (tw:(unit$)) AND (tw:((halt$ OR limit$ OR delay$) AND (admit$ or admis$))) AND (tw:(infection$ OR virus$ OR pathogen$ OR microb$ OR mycobacteri$ OR hai OR nosocomial)) AND (instance:"regional") AND (db:("LILACS")) | 29 |
| (tw:(unit$)) AND (tw:((halt$ OR limit$ OR delay$) AND (admit$ or admis$))) AND (tw:(tuberculosis OR Klebsiella)) AND (instance:"regional") AND ( db:("LILACS")) | 4 |
| (tw:(unit$)) AND (tw:((halt$ OR limit$ OR delay$) AND (admit$ or admis$))) AND (tw:(measles OR mdn1 OR "midasin homolog" OR esbl OR "Extended Spectrum Beta Lactamase" OR "Clostridium difficile" OR norovirus OR calicvir$ OR Rotavirus OR kpc OR Coronavirus OR "Middle East respiratory syndrome" OR Adenovir$ OR Mastadenovirus OR "severe acute respiratory syndrome" OR sars OR bocavirus OR pneumovirus OR parvovirus OR parechovirus OR orthomyxovirus OR gastroenteritis)) AND (instance:"regional") AND ( db:("LILACS")) | 0 |
| (tw:(ward$) AND (close$ OR closure$)) AND (tw:(outbreak$ OR disease$ transmi$ OR spread$ OR coloniz$ OR colonis$)) (instance:"regional") AND (db: ("LILACS")) | 16 |
| (tw:(ward$) AND (close$ OR closure$)) AND (tw:(infection$ OR virus$ OR pathogen$ OR microb$ OR mycobacteri$ OR hai OR nosocomial)) AND (instance:"regional") AND (db:("LILACS")) | 9 |
| (tw:(ward$) AND (close$ OR closure$)) AND (tw:(staphylococc$ OR entero$ OR gastroenteritis)) AND (instance:"regional") AND ( db:("LILACS")) | 4 |
| (tw:(ward$) AND (close$ OR closure$)) AND (tw:(influenza OR flu OR mdn1 OR midasin homolog OR esbl OR "Extended Spectrum Beta Lactamase" OR "Clostridium difficile" OR Klebsiella OR diarrhea OR "respiratory syncytial" OR norovirus OR calicvir$ OR Rotavirus OR kpc OR Coronavirus OR "Middle East respiratory syndrome" OR Adenovir$ OR Mastadenovirus OR "severe acute respiratory syndrome" OR sars OR bocavirus OR pneumovirus OR parvovirus OR parechovirus OR orthomyxovirus OR tuberculosis OR measles)) AND (instance:"regional") AND ( db:("LILACS")) | 0 |
| (tw:(ward$)) AND (tw:((halt$ OR limit$ OR delay$) AND (admit$ or admis$))) AND (instance:"regional") AND (db: ("LILACS")) | 13 |
| (tw:(hospital) AND (close$ OR closure$)) AND (tw:(outbreak$ OR disease$ transmit$ OR spread$ OR coloniz$ or colonis$)) AND (tw:(infection$ OR virus$ OR pathogen$ OR microb$ OR mycobacteri$ OR hai OR nosocomial)) AND (instance:"regional") AND (db:("LILACS")) | 211 |
| (tw:(hospital) AND (close$ OR closure$)) AND (tw:(tuberculosis OR influenza OR flu OR staphylococc$ OR Klebsiella OR diarrhea OR gastroenteritis OR "respiratory syncytial")) AND (instance:"regional") AND ( db:("LILACS")) | 59 |
| (tw:(hospital) AND (close$ OR closure$)) AND (tw:(mdn1 OR "midasin homolog" OR esbl OR "Extended Spectrum Beta Lactamase" OR "Clostridium difficile" OR norovirus OR calicvir$ OR Rotavirus OR kpc OR Coronavirus OR "Middle East respiratory syndrome" OR Adenovir$ OR Mastadenovirus OR "severe acute respiratory syndrome" OR sars OR bocavirus OR pneumovirus OR metapneumovirus OR entero$ OR parvovirus OR parechovirus OR orthomyxovirus OR measles)) AND (instance:"regional") AND ( db:("LILACS")) | 0 |
| (tw:(hospital)) AND (tw:((halt$ OR limit$ OR delay$) AND (admit$ or admis$))) AND (tw:(outbreak$ OR disease$ transmit$ OR spread$ OR coloniz$ OR colonis$)) AND (instance:"regional") AND ( db:("LILACS")) | 156 |
| (tw:(hospital)) AND (tw:((halt$ OR limit$ OR delay$) AND (admit$ or admis$))) AND (tw:(infection$ OR virus$ OR pathogen$ OR microb$ OR mycobacteri$ OR hai OR nosocomial)) AND (instance:"regional") AND (db:("LILACS")) | 69 |
| (tw:(hospital)) AND (tw:((halt$ OR limit$ OR delay$) AND (admit$ or admis$))) AND(tw:(tuberculosis OR staphylococc$ OR entero$ OR influenza OR esbl OR Rotavirus OR Klebsiella OR diarrhea)) AND (instance:"regional") AND ( db:("LILACS")) | 24 |
| (tw:(hospital)) AND (tw:((halt$ OR limit$ OR delay$) AND (admit$ or admis$))) AND (tw:(flu OR mdn1 OR "midasin homolog" OR "Extended Spectrum Beta Lactamase" OR "Clostridium difficile" OR norovirus OR calicvir$ OR kpc OR Coronavirus OR "Middle East respiratory syndrome" OR Adenovir$ OR Mastadenovirus OR "severe acute respiratory syndrome" OR sars OR "respiratory syncytial" OR bocavirus OR pneumovirus OR metapneumovirus OR parvovirus OR parechovirus OR orthomyxovirus OR gastroenteritis OR measles)) AND (instance:"regional") AND ( db:("LILACS")) | 0 |
| (tw:(bay$) AND (close$ OR closure$)) AND (tw:(outbreak$ OR disease$ transmit$ OR spread$ OR coloniz$ or colonis$)) AND (instance:"regional") AND (db:("LILACS")) | 8 |
| (tw:(bay$) AND (close$ OR closure$)) AND (tw:(infection$ OR virus$ OR pathogen$ OR microb$ OR mycobacteri$ OR hai OR nosocomial)) AND (instance:"regional") AND (db:("LILACS")) | 11 |
| (tw:(bay$) AND (close$ OR closure$)) AND (tw:(tuberculosis OR entero$)) AND (instance:"regional") AND ( db:("LILACS")) | 3 |
| (tw:(bay$) AND (close$ OR closure$)) AND (tw:(mdn1 OR "midasin homolog" OR esbl OR "Extended Spectrum Beta Lactamase" OR "Clostridium difficile" OR norovirus OR calicvir$ OR Rotavirus OR kpc OR Coronavirus OR "Middle East respiratory syndrome" OR Adenovir$ OR Mastadenovirus OR "severe acute respiratory syndrome" OR sars OR bocavirus OR pneumovirus OR metapneumovirus OR parvovirus OR parechovirus OR orthomyxovirus OR measles OR influenza OR flu OR staphylococc$ OR Klebsiella OR diarrhea OR gastroenteritis OR "respiratory syncytial")) AND (instance:"regional") AND ( db:("LILACS")) | 0 |
| (tw:(bay$)) AND (tw:((halt$ OR limit$ OR delay$) AND (admit$ or admis$))) AND (instance:"regional") AND (db:("LILACS")) | 2 |
| (tw:(partial$) AND (close$ OR closure$)) AND (tw:(outbreak$ OR disease$ transmit$ OR spread$ OR coloniz$ or colonis$)) AND (instance:"regional") AND ( db:("LILACS")) | 55 |
| (tw:(partial$) AND (close$ OR closure$)) AND (tw:(infection$ OR virus$ OR pathogen$ OR microb$ OR mycobacteri$ OR hai OR nosocomial)) AND (instance:"regional") AND (db:("LILACS")) | 30 |
| (tw:(partial$) AND (close$ OR closure$)) AND (tw:(tuberculosis OR staphylococc$ OR entero$)) AND (instance:"regional") AND ( db:("LILACS")) | 5 |
| (tw:(partial$) AND (close$ OR closure$)) AND (tw:(influenza OR flu OR OR mdn1 OR "midasin homolog" OR esbl OR "Extended Spectrum Beta Lactamase" OR "Clostridium difficile" OR norovirus OR calicvir$ OR Rotavirus OR Klebsiella OR kpc OR Coronavirus OR "Middle East respiratory syndrome" OR diarrhea OR Adenovir$ OR Mastadenovirus OR "severe acute respiratory syndrome" OR sars OR "respiratory syncytial" OR bocavirus OR pneumovirus OR metapneumovirus OR parvovirus OR parechovirus OR orthomyxovirus OR gastroenteritis OR measles)) AND (instance:"regional") AND ( db:("LILACS")) | 0 |
| (tw:(cohorting)) AND (tw:(outbreak$ OR disease$ transmit$ OR spread$ OR coloniz$ or colonis$)) AND (instance:"regional") AND ( db:("LILACS")) | 0 |
| (tw:(cohorting)) AND (tw:(infection$ OR virus$ OR pathogen$ OR microb$ OR mycobacteri$ OR hai OR nosocomial)) AND (instance:"regional") AND (db:("LILACS")) | 0 |
| (tw:(cohorting)) AND (tw:(tuberculosis OR staphylococc$ OR entero$ OR influenza OR flu OR OR mdn1 OR "midasin homolog" OR esbl OR "Extended Spectrum Beta Lactamase" OR "Clostridium difficile" OR norovirus OR calicvir$ OR Rotavirus OR Klebsiella OR kpc OR Coronavirus OR "Middle East respiratory syndrome" OR diarrhea OR Adenovir$ OR Mastadenovirus OR "severe acute respiratory syndrome" OR sars OR "respiratory syncytial" OR bocavirus OR pneumovirus OR metapneumovirus OR parvovirus OR parechovirus OR orthomyxovirus OR gastroenteritis OR measles)) AND (instance:"regional") AND ( db:("LILACS")) | 0 |
| (tw:(department$)) AND (tw:(close$ OR closure$)) AND (tw:(outbreak$ OR disease$ transmi$ OR spread$ OR coloniz$ OR colonis$)) AND (instance:"regional") AND ( db:("LILACS")) | 65 |
| (tw:(department$)) AND (tw:(close$ OR closure$)) AND (tw:(infection$ OR virus$ OR pathogen$ OR microb$ OR mycobacteri$ OR hai OR nosocomial)) AND (instance:"regional") AND (db:("LILACS")) | 0 |
| (tw:(department$)) AND (tw:(close$ OR closure$)) AND (tw:(tuberculosis OR staphylococc$ OR entero$ OR influenza OR flu OR OR mdn1 OR "midasin homolog" OR esbl OR "Extended Spectrum Beta Lactamase" OR "Clostridium difficile" OR norovirus OR calicvir$ OR Rotavirus OR Klebsiella OR kpc OR Coronavirus OR "Middle East respiratory syndrome" OR diarrhea OR Adenovir$ OR Mastadenovirus OR "severe acute respiratory syndrome" OR sars OR "respiratory syncytial" OR bocavirus OR pneumovirus OR metapneumovirus OR parvovirus OR parechovirus OR orthomyxovirus OR gastroenteritis OR measles)) AND (instance:"regional") AND ( db:("LILACS")) | 0 |
| (mh:("infection control")) AND (tw:(close$ OR closure$)) AND (instance:"regional") AND (db: ("LILACS")) | 8 |
| (mh:("cross infection")) AND (tw:(close$ OR closure$)) AND (instance:"regional") AND (db: ("LILACS")) | 49 |
| (mh:("health facility closure")) AND (instance:"regional") AND ( db:("LILACS")) | 14 |

**IndMed Lars Strategy- Search run on February 3**

| Searches | Results |
| --- | --- |
| (facility or facilities) AND (close or closes or closed or closure or closures) AND (infectious disease or infectious diseases or communicable disease or communicable diseases or disease transmission or pathogen or pathogens or nosocomial or hai or hospital acquired or hospital associated or outbreak or outbreaks) | 0 |
| (facility or facilities) AND (close or closes or closed or closure or closures) AND (influenza or flu or mdn1 or esbl or Clostridium difficile or Rotavirus or Klebsiella or kpc or Coronavirus or Middle East respiratory syndrome or diarrhea or mrsa or mssa) | 1 |
| (facility or facilities) AND (close or closes or closed or closure or closures) AND (severe acute respiratory syndrome or sars or respiratory syncytial or bocavirus or pneumovirus or metapneumovirus or parvovirus or parechovirus or orthomyxovirus or calicvirus or adenovirus or mastadenovirus) | 0 |
| (facility or facilities) AND (close or closes or closed or closure or closures) AND (tuberculosis or measles or staphylococcus or norovirus or enterococcus or enterovirus or gastroenteritis) | 3 |
| (ward or wards) AND (close or closes or closed or closure or closures) | 13 |
| (bay or bays) AND (close or closes or closed or closure or closures) | 0 |
| (department or departments) AND (close or closes or closed or closure or closures) AND (infectious disease or infectious diseases or communicable disease or communicable diseases or disease transmission or pathogen or pathogens or nosocomial or hai or hospital acquired or hospital associated or outbreak or outbreaks) | 10 |
| (department or departments) AND (close or closes or closed or closure or closures) AND (influenza or flu or mdn1 or esbl or Clostridium difficile or Rotavirus or Klebsiella or kpc or Coronavirus or Middle East respiratory syndrome or diarrhea or mrsa or mssa) | 7 |
| (department or departments) AND (close or closes or closed or closure or closures) AND (severe acute respiratory syndrome or sars or respiratory syncytial or bocavirus or pneumovirus or metapneumovirus or parvovirus or parechovirus or orthomyxovirus or calicvirus or adenovirus or mastadenovirus) | 0 |
| (department or departments) AND (close or closes or closed or closure or closures) AND (tuberculosis or measles or staphylococcus or norovirus or enterococcus or enterovirus or gastroenteritis) | 41 |
| (hospital or hospitals) AND (close or closes or closed or closure or closures) AND (infectious disease or infectious diseases or communicable disease or communicable diseases or disease transmission or pathogen or pathogens or nosocomial or hai or hospital acquired or hospital associated or outbreak or outbreaks) | 11 |
| (hospital or hospitals) AND (close or closes or closed or closure or closures) AND (influenza or flu or mdn1 or esbl or Clostridium difficile or Rotavirus or Klebsiella or kpc or Coronavirus or Middle East respiratory syndrome or diarrhea or mrsa or mssa ) | 5 |
| (hospital or hospitals) AND (close or closes or closed or closure or closures) AND (severe acute respiratory syndrome or sars or respiratory syncytial or bocavirus or pneumovirus or metapneumovirus or parvovirus or parechovirus or orthomyxovirus or calicvirus or adenovirus or mastadenovirus) | 0 |
| (hospital or hospitals) AND (close or closes or closed or closure or closures) AND (tuberculosis or measles or staphylococcus or norovirus or enterococcus or enterovirus or gastroenteritis) | 23 |
| (unit or units) AND (close or closes or closed or closure or closures) AND (infectious disease or infectious diseases or communicable disease or communicable diseases or disease transmission or pathogen or pathogens or nosocomial or hai or hospital acquired or hospital associated or outbreak or outbreaks) | 0 |
| (unit or units) AND (close or closes or closed or closure or closures) AND (influenza or flu or mdn1 or esbl or Clostridium difficile or Rotavirus or Klebsiella or kpc or Coronavirus or Middle East respiratory syndrome or diarrhea or mrsa or mssa) | 1 |
| (unit or units) AND (close or closes or closed or closure or closures) AND (severe acute respiratory syndrome or sars or respiratory syncytial or bocavirus or pneumovirus or metapneumovirus or parvovirus or parechovirus or orthomyxovirus or calicvirus or adenovirus or mastadenovirus) | 0 |
| (unit or units) AND (close or closes or closed or closure or closures) AND (tuberculosis or measles or staphylococcus or norovirus or enterococcus or enterovirus or gastroenteritis) | 3 |
| (partial or partly or in part or in parts) AND (close or closes or closed or closure or closures) AND (infectious disease or infectious diseases or communicable disease or communicable diseases or disease transmission or pathogen or pathogens or nosocomial or hai or hospital acquired or hospital associated or outbreak or outbreaks) | 1 |
| (partial or partly or in part or in parts) AND (close or closes or closed or closure or closures) AND (influenza or flu or mdn1 or esbl or Clostridium difficile or Rotavirus or Klebsiella or kpc or Coronavirus or Middle East respiratory syndrome or diarrhea or mrsa or mssa) | 2 |
| (partial or partly or in part or in parts) AND (close or closes or closed or closure or closures) AND (severe acute respiratory syndrome or sars or respiratory syncytial or bocavirus or pneumovirus or metapneumovirus or parvovirus or parechovirus or orthomyxovirus or calicvirus or adenovirus or mastadenovirus) | 0 |
| (partial or partly or in part or in parts) AND (close or closes or closed or closure or closures) AND (tuberculosis or measles or staphylococcus or norovirus or enterococcus or enterovirus or gastroenteritis) | 0 |
| (cohorting) AND (close or closes or closed or closure or closures) | 0 |
| (delay or delayed or limit or limited or halt or halted) AND (admit or admission) AND (infectious disease or infectious diseases or communicable disease or communicable diseases or disease transmission or pathogen or pathogens or nosocomial or hai or hospital acquired or hospital associated or outbreak or outbreaks) | 1 |
| (delay or delayed or limit or limited or halt or halted) AND (admit or admission) AND (influenza or flu or mdn1 or esbl or Clostridium difficile or Rotavirus or Klebsiella or kpc or Coronavirus or Middle East respiratory syndrome or diarrhea or mrsa or mssa) | 3 |
| (delay or delayed or limit or limited or halt or halted) AND (admit or admission) AND (severe acute respiratory syndrome or sars or respiratory syncytial or bocavirus or pneumovirus or metapneumovirus or parvovirus or parechovirus or orthomyxovirus or calicvirus or adenovirus or mastadenovirus) | 0 |
| (delay or delayed or limit or limited or halt or halted) AND (admit or admission) AND (tuberculosis or measles or staphylococcus or norovirus or enterococcus or enterovirus or gastroenteritis) | 7 |
